# Exploring the nature of interaction between shiga toxin producing *Escherichia coli* (STEC) and free-living amoeba - *Acanthamoeba* sp

**DOI:** 10.3389/fcimb.2022.926127

**Published:** 2022-09-09

**Authors:** Margherita Montalbano Di Filippo, Arianna Boni, Paola Chiani, Manuela Marra, Maria Carollo, Lucrezia Cristofari, Fabio Minelli, Arnold Knijn, Stefano Morabito

**Affiliations:** ^1^ Department of Food Safety, Nutrition and Veterinary Public Health, Istituto Superiore di Sanità, Rome, Italy; ^2^ Core Facilities, Istituto Superiore di Sanità, Rome, Italy

**Keywords:** Shiga toxin producing *Escherichia coli* (STEC), free- living amoebae (FLA), *E. coli* O157, food-safety, zoonosis

## Abstract

Free-living amoebae (FLA) are widely distributed protozoa in nature, known to cause severe eye infections and central nervous system disorders. There is growing attention to the potential role that these protozoa could act as reservoirs of pathogenic bacteria and, consequently, to the possibility that, the persistence and spread of the latter may be facilitated, by exploiting internalization into amoebae. Shiga toxin-producing strains of *Escherichia coli* (STEC) are zoonotic agents capable of causing serious diseases, such as hemorrhagic colitis (HC) and hemolytic uremic syndrome (HUS). Cattle represent the main natural reservoir of STEC, which are frequently found also in other domestic and wild ruminants, often without causing any evident symptoms of disease. The aspects related to the ecology of STEC strains in animal reservoirs and the environment are poorly known, including the persistence of these microorganisms within niches unfavorable to survival, such as soils or waters. In this study we investigated the interaction between STEC strains of serotype O157: H7 with different virulence gene profiles, and a genus of a wild free-living amoeba, *Acanthamoeba* sp. Our results confirm the ability of STEC strains to survive up to 20 days within a wild *Acanthamoeba* sp., in a quiescent state persisting in a non-cultivable form, until they reactivate following some stimulus of an unknown nature. Furthermore, our findings show that during their internalization, the *E. coli* O157 kept the set of the main virulence genes intact, preserving their pathogenetic potential. These observations suggest that the internalization in free-living amoebae may represent a means for STEC to resist in environments with non-permissive growth conditions. Moreover, by staying within the protozoa, STEC could escape their detection in the vehicles of infections and resist to the treatments used for the disinfection of the livestock environment.

## Introduction

Free-living amoebae (FLA) are ubiquitous and widely distributed Protozoa commonly found in terrestrial and aquatic environments where they feed on bacteria, algae, and fungi.

These organisms have also been isolated in various man-made constructions such as drinking water supplies, hospital water networks, eyewash stations, swimming pools, spas, homes, dental unit’s waterlines, cooling towers and wastewater water plants. Despite their wide distribution, only some genera of FLA are known to be potentially pathogenic to humans.

FLA are characterized by two life cycle stages: the trophozoite and the resistant cyst. The former represents the actively feeding stage, preying on bacteria, algae, viruses, yeast (Siddiqui and Khan, 2012). Encystment is triggered by adverse environmental conditions: indeed, on cystic stage, the amoebae are preserved against food shortage, high/low temperatures, pH extremes, gamma and UV radiation, and chemicals used for disinfection in health care settings and drinking water production (Nataro and Kaper, 1998; Tozzi et al., 2003; Caprioli et al., 2005; Khan, 2006; Loret and Greub, 2010; Thomas et al., 2010; Siddiqui and Khan, 2012).

In addition to their pathogenicity, there is growing attention about the potential role that FLA could play as reservoirs of pathogenic bacteria and, consequently, to the possibility that, their persistence and spread may be facilitated, by exploiting internalization into amoebae. The relationship between bacteria and amoeba may serve (i) to protect bacteria from hostile conditions; and (ii) to support bacteria to adapt to persistence in mammalian phagocytic cells, indicating that amoeba-bacteria relationships are involved in complex interactions (Greub and Raoult, 2004). In addition, the ability of bacteria to resist to grazing by amoeba may have led to their evolution to cause human diseases, i.e., evade human immune cells such as macrophages (Greub and Raoult, 2004). Recent studies have shown FLA could represent a niche for many human bacterial pathogens (Scheid, 2018), including food-related pathogens (Vaerewijck et al., 2014; Lambrecht et al., 2015), to survive and replicate in, therefore representing an efficient vehicle to increase the persistence and diffusion of amoeba resistant bacteria (ARBs) in the environment.

*Acanthamoeba* is a free-living amoeba that plays an important role in controlling bacterial populations in a variety of terrestrial environments, and it serves as a model for the study of protozoa–bacteria predator–prey interactions (Henriquez et al., 2021). It is one of the most common FLA genera found in soil and water samples (Page, 1988) having a cosmopolitan distribution and a wide variety of habitats. Its wide distribution in nature brings frequently humans in contact with this amoeba, and evidence is found in the extensive presence of related antibodies in human and animal populations.

In addition to causing severe ocular and brain infections in humans, *Acanthamoeba* has been shown to harbor many bacterial, viral, and fungal species, although not always to their own advantage (Rayamajhee et al., 2021).

*Escherichia coli* O157:H7 is considered the archetype Shiga toxin encoding *E. coli* (STEC) strain (Gyles, 2007). STEC are characterized by the production of Shiga toxins (stx), potent cytotoxins that inhibit the protein synthesis within eukaryotic cells (Melton-Celsa, 2014), which are encoded by the *stx* genes harbored on lysogenic bacteriophages. Stxs are the major virulence factor of STEC and comprise a family of structurally related cytotoxins with similar biological activity. Two main types of *stx* genes have been described, *stx1* and *stx2*, which, as of now, have been subtyped in three and eleven subtypes, respectively (Scheutz et al., 2012; Lacher et al., 2016; Bai et al., 2018; Yang et al., 2019).

STEC are zoonotic pathogens, causing foodborne infections with symptoms ranging from mild diarrhea to hemolytic uremic syndrome (HUS), sometimes with fatal outcomes (Caprioli et al., 2005). Infection is transmitted to humans through the ingestion of contaminated food, but person to person transfer and direct contact with infected animals or contaminated environment, especially water is also reported (Caprioli et al., 2005).

Cattle represent the main natural reservoir of STEC, which are, however, frequently found also in other domestic and wild ruminants, often without causing any evident symptoms of disease (Caprioli et al., 2005).

The aspects related to the ecology of STEC strains in animal reservoirs and the environment, as well as the related biological mechanisms are poorly known, including the persistence of these microorganisms within niches unfavorable to survival, such as soils or waters.

In the present work, we used a combined approach to investigate the nature of interaction between STEC and the free-living amoeba *Acanthamoeba*.

We (i) investigated the internalization ability of a panel of STEC O157: H7, with different virulence gene profiles, in a field isolate of *Acanthamoeba* sp.; (ii) determined whether the bacterium remains quiescent or in a state of active growth within the protozoan; (iii) studied the integrity and genomic stability of the STEC strains internalized in *Acanthamoeba* sp. in comparison with the same isolates grown in permissive culture conditions.

## Materials and methods

### Amoebal strain and culture conditions

An environmental isolate of *Acanthamoeba* sp. belonging to the T4 genotype, isolated from thermal waters (T = 40°C), was kindly obtained from Dr. David Di Cave and Dr. Federica Berrilli (Parasitology Unit, University of Rome “Tor Vergata”, Italy). The amoebae were grown without shaking in proteose peptone-yeast-glucose (PYG – 0,75%, w/v, proteose peptone; 0,75%, w/v, yeast extract; 1,5%, w/v, glucose) medium as monolayers in 75 cm^2^ tissue culture flasks at 37°C and subsequently, were sub-cultured weekly by tapping flasks to detach cells before diluting 1:5 in fresh medium (Berk and Garduno, 2012). Stationary phase (5-10 days) cultures were used throughout this study (95% trophozoite form).

### Bacterial strains and culture conditions

Three STEC O157: H7 strains from the culture collection at National Reference Laboratory for *E. coli* (Istituto Superiore di Sanità, Italy) and a commensal strain of *E. coli* used as a non-pathogenic control were used in this study (**Table 1**).

**Table 1:** Characteristics of bacterial strains used in this study.

Strain	Serotype	Virulence profile	Source
EDL933 (STEC)	O157:H7	*eae, stx1a, stx2a*	Human stool (HUS)
ED605 (STEC)	O157:H7	*eae, stx1a*	Human stool (HUS)
ED638 (STEC)	O157:H7	*eae, stx2c*	Bovine stool
ECORI	O144:H4	–	Human stool

All bacteria were grown in Luria–Bertani (LB) broth overnight. Prior to the intracellular survival assays, all the strains were checked for their ability to grow in presence of the antibiotic used in the internalization experiments. For co-culture experiments, the overnight cultures of bacteria in LB broth were refreshed with the ratio of 1:10 into fresh LB broth and incubated at 37℃ with continuous shaking until the absorbance at 590 nm reached 0.5 to 0.6.

### Intracellular survival assays

To study STEC interactions with *Acanthamoeba* sp., intracellular survival assays were performed in triplicates, as previously described (Jung et al., 2007; Jacquier et al., 2013; Yousuf et al., 2013). Briefly, free-living amoebae were inoculated in 48-well plates in PYG medium. The plates were incubated at 37℃ for 24-48 h to obtain confluent cultures (at least 50%). After this incubation, media were aspirated, and wells were washed once with phosphate buffered saline (PBS). Subsequently, *E. coli* strains were added (10^6^ CFU ml^-1^), and plates incubated for 120 min at room temperature. Following this incubation, gentamicin (150 μg/ml) was added to each well to kill extracellular bacteria and eventually the plates were incubated at 37°C for 25 days. At 12 h, 24 h, 72 h, 6 days, 10 days, 15 days, 20 days and 25 days intervals, the medium in the single wells was carefully checked for bacterial contamination and for amoeba integrity. The protozoa were eventually washed three times with PBS and the amoebae lysed by adding SDS (0.5% final conc.) to each well for 40 min and the presence of live bacteria was assessed by plating onto MacConkey agar plates, with and without sorbitol and the resulting CFU enumerated. Randomly picked single bacterial colonies were used as template in a specific PCR for the detection of *stx1*, *stx2* and *eae* genes (Paton and Paton, 1998).

### Analysis of the expression of *rpoS* gene

The resuscitation of several species of Viable But Not Culturable (VBNC) bacteria upon being internalized by the amoebae has been documented (Steinert et al., 1997; García et al., 2007; Epalle et al., 2014; Casini et al., 2018; Dey et al., 2020). The biological reason of this shift is unknown and largely unexplored. To verify whether the lack of bacterial growth registered at certain sampling times during the invasion experiment was due to the present of VBNC forms of the internalized strains, we conducted an analysis of the of *rpoS* gene expression. This gene encodes a conserved alternative sigma factor that regulates the expression of many stress response genes in *E. coli.* The expression of *rpoS* gene was analyzed using the total RNA prepared from the amoebic SDS (0.5%) lysates, sampled in a replicated intracellular assay were the protozoa were sampled at 24 h, 72 h and 10 days. Additionally, the total RNA was extracted from one ml of overnight broth cultures grown at 37°C of each *E. coli* strains used in this internalization experiment to be used as calibrator in the expression experiments. The RNA was extracted using the Norgen RNA/Protein Purification Kit, according to the manufacturer’s instructions. In detail, 1µg of RNA was used for DNA removal and retro-transcription with the QuantiTect Reverse Transcription (Qiagen, Germantown, MD, USA). Two µl of the cDNA solutions were used in Real Time PCR reactions targeting *rpoS* gene in 40 cycles of a two steps thermal profile (10 s at 95°C and 1 min at 55°C) using the following primers and probes: *rpoS*_FWD: TTCGTTTGCCGATTCACATC; *rpoS*_REV: TCTCTTCCGCACTTGGTTCA; *rpoS*_probe: TTACCTGCGAACAGCAC (Liu et al., 2010). The transcription of the housekeeping gene *gapA* (Fitzmaurice et al., 2004) was measured and used to normalize the amount of template used, while the level of transcription recorded in the RNA preparations from the strains grown in broth cultures was used as calibrator to calculate the relative fold change in gene expression by the 2^−ΔΔCT^ threshold cycle (C_T_) method (Schmittgen, 2001).

### Whole genome sequencing of the internalized *E. coli* strains

All bacterial strains internalized within *Acanthamoeba* and grown on the plates were subjected to whole genome sequencing (WGS) to investigate their genome’s integrity and stability. Total DNA was extracted from a 2 mL overnight culture of each strain grown in TSB at 37°C, with the GRS Genomic DNA Kit Bacteria (GRISP Research Solutions, Porto, Portugal). The genomes’ sequences were produced with an Ion Torrent S5 sequencing platform (Thermo Fisher Scientific, MA, USA). Libraries of about 400 bp from 100 ng of total DNA were prepared using the NEBNext^®^ Fast DNA Fragmentation & Library Prep Set for Ion Torrent™ (#E6285L, New England BioLabs, MA, USA). Following preparation, library qualities and amounts were assessed using the Tape Station System 4200 with the High Sensitivity D1000 Reagents Kit (Agilent Technologies #5067-5585). Libraries were enriched on the Ion Chef System using the Ion 510™ & 520™ & 530™ Kit (#A34018, #A27754, #A27755, Ion Chef Supplies/Solutions, Thermo Fisher Scientific, MA, USA), and eventually subjected to sequencing.

### Genomic characterization of the strains used in the internalization experiments

The bioinformatic analyses were carried out using the tools present in the Galaxy public server ARIES (https://www.iss.it/site/aries) (Knijn et al., 2020). The virulence genes content of the genomes was determined with the Patho_typing tool (https://github.com/B-UMMI/patho_typing) developed by the INNUENDO project (Llarena et al., 2018) using the *E. coli* virulence genes database (Joensen et al., 2014). Antimicrobial resistance genes were detected through ABRicate v0.8.13 using the ResFinder database (https://github.com/tseemann/abricate). Phylogenomics analysis was performed by determining the core genome MLST (cgMLST) using the chewBBACA tool (Silva et al., 2018) and the scheme developed by the INNUENDO project, which comprises 2,360 loci in total (Llarena et al., 2018; Silva et al., 2018). The distances between strains were calculated by pairwise comparison of the allelic profiles through the chewTree tool (https://www.iss.it/site/aries). The pairwise comparison was considered reliable when >80% of loci for each sample were assigned to an allele. For each pair of samples, the alleles which were not found, only partially found, or not correctly assigned to any locus were excluded from the analysis, as previously described (Gigliucci et al., 2021). The resulting dendrogram was visualized and modified using iTOL (https://itol.embl.de/ - Letunic and Bork, 2007).

## Results

### *E. coli* survival within *Acanthamoeba* sp. in view of long-term interactions

Our findings revealed that all *E. coli* strains tested were internalized and remained viable inside *Acanthamoeba* sp. for the entire length of the experiments, with various growth rates, depending on the strain (**Table 2**). In addition, *Acanthamoeba* sp. remained intact throughout all the incubation period without added nutrients at 37°C as assessed by microscope observation. All internalized STEC strains maintained their *stx1*; *stx2*; *eae* virulence genes profile (see **Table 1** – Materials and Methods section) during all time (12h – 25 days). Interestingly, STEC strains did not show growth on plate at 20 days, (**Table 2**) whereas an intense bacterial growth was observed at 25 days (**Table 2**).

**Table 2:** *E. coli* (CFU) intracellular of *Acanthamoeba* sp. reported in triplicate.

	EDL933 (O157:H7)			ED605 (O157:H7)			ED638 (O157:H7)			ECORI (O144:H4)		
**12 hours**	100 CFU	70 CFU	>100 CFU	1 CFU	0 CFU	0 CFU	1 CFU	0 CFU	2 CFU	1 CFU	22 CFU	6 CFU
	Median: 97 CFU SD: 25.16			1 CFU SD: 0.57			Median: 1 CFU SD: 1			Median: 10 CFU SD: 10.96		
**24 hours**	>100 CFU	100 CFU	50 CFU	100 CFU	>100 CFU	70 CFU	100 CFU	40 CFU	53 CFU	100 CFU	>100 CFU	25 CFU
	Median: 90 CFU SD: 36.05			Median: 96 CFU SD: 25.16			Median: 64 CFU SD: 31.56			Median: 81 CFU SD: 50.08		
**72 hours**	0 CFU	2 CFU	0 CFU	>100 CFU	30 CFU	80 CFU	0 CFU	0 CFU	4 CFU	0 CFU	5 CFU	3 CFU
	2 CFU SD: 1.15			Median: 77 CFU SD: 45.09			4 CFU SD: 2.30			3 CFU SD: 2.51		
**6 days**	0 CFU	0 CFU	1 CFU	1 CFU	0 CFU	5 CFU	14 CFU	0 CFU	8 CFU	100 CFU	100 CFU	45 CFU
	1 CFU SD: 0.57			Median: 2 CFU SD: 2.64			Median: 7 CFU SD: 7.02			Median: 82 CFU SD: 31.75		
**10 days**	100 CFU	80 CFU	>100 CFU	30 CFU	10 CFU	7 CFU	100 CFU	60 CFU	>100 CFU	>100 CFU	>100 CFU	70 CFU
	Median: 100 CFU SD: 20			Median: 16 CFU SD: 12.50			Median: 93 CFU SD: 30.55			Median: 103 CFU SD: 28.86		
**15 days**	23 CFU	4 CFU	8 CFU	0 CFU	1 CFU	0 CFU	0 CFU	1 CFU	0 CFU	100 CFU	80 CFU	>100 CFU
	Median: 12 CFU SD: 10.01			1 CFU SD: 0.57			1 CFU SD: 0.57			Median: 100 CFU SD: 20		
**20 days**	0 CFU	0 CFU	1 CFU	0 CFU	0 CFU	0 CFU	0 CFU	0 CFU	0 CFU	100 CFU	100 CFU	>100 CFU
	1 CFU SD: 0.57			0 CFU SD: 0			0 CFU SD: 0			Median: 106 CFU SD: 11.55		
**25 days**	70 CFU	30 FCU	15 CFU	>100 CFU	30 CFU	100 CFU	>100 CFU	23 CFU	44 CFU	1 CFU	10 CFU	7 CFU
	Median: 38 CFU SD: 28.43			Median: 83 CFU SD: 47.25			Median: 62 CFU SD: 51.03			Median: 6 CFU SD: 4.58		

The Median and the Standard Deviations (SD) of the bacterial counts deriving from triplicate intracellular assays are calculated.

### The gene *rpoS* is expressed in sampling times that did not show bacterial growth

**Figure 1:**
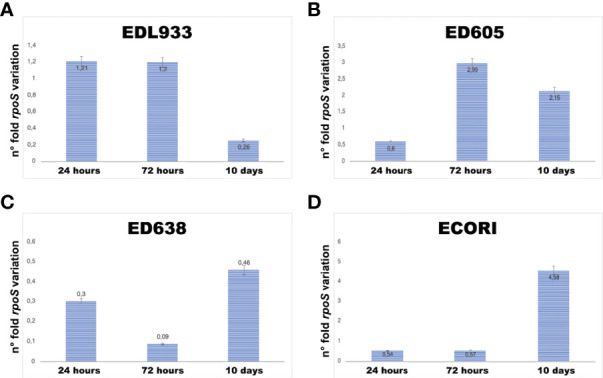
*rpoS*’ gene transcription profiles **(A–D)**. The mRNA of the housekeeping gene *gapA* was used normalize the template (normalizator), while the *rpoS* mRNA of the same strains grown in broth at permissive conditions was used to calculate the relative fold change in gene expression by the 2^−ΔΔCT^ threshold cycle (C_T_) method (calibrator).

As we could not see growth of bacterial cells in some sampling times (**Table 2**), an additional short-time internalization experiment was carried out to investigate the expression of *rpoS* gene in the internalized bacteria where we sampled the wells at 24 and 72 hours and at 10 days (**Table 3**). The *rpoS* is a conserved alternative sigma factor that regulates the expression of many stress response genes and is generally used a selective marker for the quantification of VBNC form of *E. coli* O157:H7 (Liu et al., 2010). In detail, our results showed that the gene *rpoS* was expressed at all sampling times, even without visible bacterial growth on the plates **(** **Table 3** and **Figure 1**). The quantitative analysis showed that the level of *rpoS* expression ranged form 0.3-fold to 4.58-fold compared with the level of expression of this gene in the same strains recovered from broth cultures **(** **Figure 1** and **Supplementary Table 1**). Strain ED638 showed the lowest levels of *rpoS* expression compared to all the other strains investigated, below the threshold of the calibrator at all sampling times (**Figure 1**). Strains EDL933 displayed the maximum level of *rpoS* transcription at 24 and 72 hours while strain ED605 produced the highest amount of *rpoS* mRNA at 72 hours, which declined slightly at ten days. The commensal *E. coli* strain showed a peculiar pattern of transcription of the *rpoS* gene reaching a peak of 4.58-fold at ten days while maintaining a basal level of transcription at the other two sampling points. The expression of *rpoS* gene in the absence of growth on plates suggests that the strains analyzed, could have remained in a quiescent state (VBNC) induced by activation of the cellular stress response.

**Table 3:** *E. coli* (CFU) intracellular of *Acanthamoeba* sp. at 24 hours, 72 hours and 10 days (short time intracellular assay).

	EDL933 (O157:H7)	ED605 (O157:H7)	ED638 (O157:H7)	ECORI (O144:H4)
**24 hours**	>100 CFU	0 CFU	0 CFU	0 CFU
**72 hours**	0 CFU	>100 CFU	0 CFU	0 CFU
**10 days**	0 CFU	0 CFU	0 CFU	0 CFU

For each sampling time, the total RNA from the amoebic SDS (0.5%) lysates was extracted.

### Genomic characterization of the internalized *E. coli* strains

The median number of contigs obtained in the generated assemblies was 128 and the median N50 value was 121115 bp. Among the three STEC strains tested, two (EDL933 and ED638) belonged to ST11 and ED605 to ST1804, while ECORI was typed as ST10. As expected, all STEC strains harbored *stx* genes, subtype *stx1a*, alone (ED605) or in combination with *stx2a* (EDL933) and subtype *stx2c* in ED638. In addition, STEC strains showed the presence of the virulence genes *eae, espA, espB* and *tir*, encoded on the LEE locus, as well as *nleB*, a non-LEE encoded effector of the Type Three Secretion System (T3SS). Regarding the antimicrobial resistance genes, all the analyzed strains were positive for the presence of *bla_EC_* genes, encoding Class C β-lactamases. Interestingly, we have noticed in the genome retrieved from one colony of the internalized non-pathogenic ECORI strain (remained in the protozoa for 6 days – see **Table 2**) the presence of one *astA* gene’ allele encoding a thermostable toxin in different pathogenic *E. coli* strains, including STEC strains (Veilleux and Dubreuil, 2006); this gene was not present in the ECORI strain prior to the internalization experiments, and the observed allele (*asta_4_ab042002*) was different from that observed in the STEC strains used in the invasion assays (*asta_8_hm099897*) (**Supplementary Table 2**). The analysis of the genome of the ECORI strain carrying the *asta_4_ab042002* allele (Acc. No. AB042002.1) showed that this sequence was part of a contig of 16.1 Kb carrying also the *fae* operon encoding the K88 fimbrial antigen of enterotoxigenic *E. coli* (Klemm, 1981), together with other genes, including some related with insertion sequences (data not shown). This contig was searched in the genome of the same ECORI sequenced before the internalization experiments using the BLASTn algorithm, with negative results.

### Phylogenomics investigation

**Figure 2:**
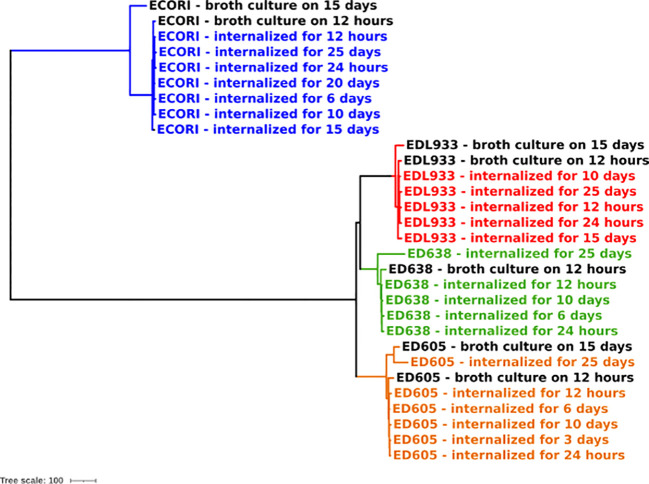
Phylogenomic analysis of *E. coli* strains internalized in *Acanthamoeba* sp. through cgMLST. The colors indicate the clade attributed to each bacterial strain.

To investigate the genomes’ integrity and stability of the *E. coli* strains internalized in *Acanthamoeba* sp., a phylogenetic analysis was performed based on cgMLST. In addition, the genomes of the same bacterial strains’ grown in broth culture for 15 days were included in the analysis. The resulting dendrogram is shown in **Figure 2**.

The analysis showed four main clades, corresponding to each bacterial strain here analyzed (EDL933 – clade, ED605 – clade, ED638 – clade and ECORI - clade). Each clade was characterized by a very low variability pointing out a higher genome stability of *E. coli* strains internalized in *Acanthamoeba* sp., comparing to same strains grown in broth for 15 days (>100 allelic differences).

The maximum number of allelic distances within clades was much lower in some clades than in others. In detail, the maximum number of allelic differences (AD) detected between the strains’ internalized at the 25 days’ time point, were 23 AD in EDL933 clade, 110 AD in ED605 clade, 42 AD in ED638 clade, and 19 AD in ECORI clade.

## Discussion

It is well known that free-living amoebae, especially *Acanthamoeba* spp., interact with many bacterial species and can play a role as their host. Despite that the mechanisms used by certain microorganisms to enter and remain vital in the amoeba are not completely clear, it is known that happens in nature and that this process may last few days or months (Rayamajhee et al., 2021). The fact that *Acanthamoeba* is a ubiquitous protozoan that can be found in many natural and/or man-made environments is relevant, making it necessary to understand the mechanisms underlying these interactions.

Several studies demonstrated that *Acanthamoeba* can interact with different pathogenic bacteria, including food-related pathogens, such as *Legionella pneumophila*, *Vibrio cholerae*, *Listeria monocytogenes*, *Salmonella* spp., *Campylobacter* sp., *Helicobacter pylori*, *Yersinia enterocolitica*, *Bacillus anthracis* and *Staphylococcus aureus* (incl. MRSA) (Balczun and Scheid, 2017; Scheid, 2018; Rayamajhee et al., 2021).

Unlike these well-studied interactions, association studies between FLA and STEC are scarce. Intracellular survival and multiplication of *E. coli* O157:H7 inside *Acanthamoeba* was first described by Barker et al., (1999). In this study, microscopical analysis revealed that several *Acanthamoeba* trophozoites contained ten or more *E. coli* cells within membrane bound vacuoles and that only in some of them bacterial digestion took place. Other authors also noted a reduced amoebal growth rate, and an increased amoebal mortality, mediated by Shiga toxins produced by *E. coli* in the cocultures. The same authors also showed that the Pho regulon was required for *E. coli* O157:H7 growth when cocultured with *Acanthamoeba*, whereas Shiga toxins as such, were not essential for bacterial survival in cocultures (Chekabab et al., 2012). The exact mechanisms by which the Pho regulon enhances intra-amoebal survival is not known, yet. Contradictory reports can be found on the Shiga toxins’ role of the internalized *E. coli* O157:H7; as a matter of fact, other studies showed that *Stxs* produced by internalized bacteria, were responsible for the killing of *Acanthamoeba* trophozoites (Chekabab et al., 2013; Arnold and Koudelka, 2014). Apart from the Pho regulon, little is known about the mechanisms involved in the interaction of STEC with FLA. Carruthers et al. (2010) described that during *E. coli* O157:H7 internalization in *Acanthamoeba*, several virulence genes, including *Stxs* and T3SS, and genes involved in the response to various stressful conditions (iron deprivation and oxidative stress) were upregulated.

In the present study, we investigated the internalization of STEC O157:H7 isolated from human disease or the animal reservoir, with different virulence gene profiles, into a wild strain of *Acanthamoeba* sp. as the host model. As *E. coli* O157:H7 encounters protozoa in the environment throughout its life cycle, we chose to use the well-studied and ubiquitous protozoan *Acanthamoeba* for this study. We observed that *Acanthamoeba* can internalize *E. coli* including STEC and that the internalized *E. coli* strains (both pathogenic and non-pathogenic) were able to survive within amoebae for at least 25 days.

These results extend the observations reported on the STEC O157 internalization to field isolates from (i) different sources and (ii) with diverse virulence genes assets. This finding is compatible with a role of *Acanthamoeba* in hosting STEC and possibly contributing to their environmental persistence, as well as in assisting STEC O157:H7 for enhanced survival during its route through the rumen of cattle. This could be similar to the *Acanthamoeba* spp. role as hosts for *V. cholerae*, *Vibrio parahaemolyticus* and *Salmonella typhi* in the aquatic environments of endemic areas (Laskowski-Arce and Orth, 2008; Abd et al., 2010; Sandstrom et al., 2010; Douesnard-Malo and Daigle, 2011; Matz et al., 2011). It’s interesting to point out that the survival within *Acanthamoeba* sp. was evidenced for STEC but also for the non-pathogenic commensal *E. coli* strain (ECORI). There are no reports describing this condition, however a non-invasive *E. coli* K-12 strain has been shown to be present intracellularly in amoebas, even if, after a short time it was lysed by amoebas, indicating *E. coli* K-12 as bacterial prey rather than an endosymbiont (Mungroo et al., 2021). We hypothesize that the strain used in this study, being a commensal in human intestine, may have acquired mechanisms allowing it to resist within the free-living amoebae. The intracellular assay’ results also showed that in different sampling times (15 days and 20 days) no bacterial growth was observed, but the vitality of the bacteria was restored at later times (25 days) (**Table 2**). This observation may be related with the internalized *E. coli* strains present in viable but non-cultivable (VBNC) forms, which is supported by the analysis of the expression of *rpoS* gene in the internalized STEC strains. As a matter of fact, our analysis exhibited the activation of the *rpoS’* transcription during the bacterial permanence inside the protozoa, (**Figure 1 A, B, D**) except for the strain ED638, (**Figure 1C**) for which the expression of this gene remained at the basal level or below throughout the whole experiment’s duration. The lack of bacterial growth in this experiment (**Table 3**), together with the detection of *rpoS* transcription, suggests that *E. coli* strains were in a state of quiescence induced by the activation of the cellular stress response.

A similar finding was described for *Aeromonas hydrophila*. For this species, the shift to the VBNC form occurs on average twenty days earlier in the presence of *Acanthamoeba* than it does alone (Rahman et al., 2008). This would reduce the window for its detection using standard techniques and increases the likelihood of underreporting its presence in water systems, eventually augmenting the chances of human infection. Interestingly, the resuscitation of several species of VBNC bacteria upon being internalized by the amoebae has been documented by several authors (Steinert et al., 1997; García et al., 2007; Epalle et al., 2014; Casini et al., 2018; Dey et al., 2019; Dey et al., 2020) as it has been observed in this study (**Table 2**). To date, the mechanism of this shift is unknown and understanding the driving forces behind these interactions could allow developing more effective culture methods that might improve the detection of pathogens in their reservoirs and certain vehicles of infections.

Due to the high plasticity of the *E. coli* genome, the maintenance of bacterial quiescence state inside the amoebae would also have been indicated by a greater genomic stability. The cgMLST performed on the genomes of internalized *E. coli* at different sampling times (**Table 2**) showed a substantial genomic stability of the STEC strains kept inside *Acanthamoeba* (**Figure 2**) which adds evidence for the survival of the internalized strains in VBNC forms.

Furthermore, the analyses revealed that the STEC strains internalized in *Acanthamoeba* maintained the virulence genes asset intact, and therefore their genetic potential for pathogenicity. Even though the STEC virulence genes are mostly present on mobile genetic elements, all the strains internalized retained the presence of the bacteriophages-related Stx-coding genes and the plasmid encoded determinants associated with the STEC pathogenicity. The mobile genetic elements, and particularly phages, are frequently lost when the bacterial cells are subjected to stress. The retention of the Stx-phages in the internalized STEC is thus interesting. Nevertheless, this observation was not surprising in the light of the activation of the *rpoS* gene. As a matter of fact, the activation of the stress response and the overexpression of this gene has been associated with lower levels of Stx-converting bacteriophages induction, reducing their lytic activation, and conferring a better survival to the bacterial cells (Imamovic et al., 2016). Finally, the finding of the presence of the *astA* gene in the genome of the non-pathogenic ECORI strain growth after 6 days of permanence inside *Acanthamoeba* was noteworthy. This gene encodes a thermostable toxin produced by different pathogenic *E. coli* strains, including STEC (Veilleux and Dubreuil, 2006). In particular, the allele identified in this strain was identical to that described in an ETEC strain O42 (Acc. No. AB042002.1) and is not present in the genome of the ECORI strain used in the internalization experiments. The sequence analysis showed that the genome of the ECORI strain isolated at 6 days from the beginning of the internalization experiment possessed at least 16.1 Kb of DNA, comprised into a single contig, absent from the original ECORI isolate and containing also other genes characteristic of the ETEC pathotype, such as the genes *faeG* and *faeE*, part of an operon encoding the K88 fimbrial antigen (Klemm, 1981). The same contig also contained genes encoding IS-associated transposases (data not shown). The gene *faeG* has been described to be present in certain plasmids of hybrid ETEC-STEC hybrid strains (Michelacci et al., 2018), suggesting that a possible mechanism for its acquisition could be mediated by a plasmid, although, at this stage we cannot exclude that other mechanisms of horizontal gene transfer may have occurred. These observations could suggest a role for the protozoan as a “genes’ reservoir”. Whether the *astA* gene identified in the ECORI strain was derived from another *E. coli* strain simultaneously present in the *Acanthamoeba* or acquired in another different way, remains to be ascertained. Whatever the mechanism was, however, our findings suggest that the acquisition of exogenous genes by bacteria during their permanence into *Acanthamoeba* is possible and strengthen the role of this amoeba as a gene melting pot (Mungroo et al., 2021). In conclusion, our findings showed that *E. coli*, including STEC, can survive inside protozoan for extended periods of time. In addition, our results support the role of *Acanthamoeba* species in being potential host reservoir for STEC, putatively conferring to these microorganisms the ability to escape their detection in the animal reservoir and the vehicles of infection, while maintaining intact their pathogenic potential. Further research is currently ongoing to elucidate the complete pattern of mechanisms and strategies used by the two organisms to come to a balanced status where both gain advantages from the co-existence.

## Data availability statement

The genome of ECORI strain internalized in the protozoa for 6 days together with the genome the of ECORI sequenced before the internalization experiments can be found in online repositories. The name of the repository and accession numbers can be found below: NCBI; PRJNA864586, SAMN30074962 - SAMN30074963.

## Author contributions

MMDF isolated and characterized the wild type amoebae strain, set up the experimental settings, and drafted the manuscript. LC performed the invasion assays and the analysis of the expression of *rpoS*. AB, PC, and FM contributed to the setup of experiments, to the scientific discussion and helped with the revision of the draft manuscript. MM and MC performed the Ion Torrent library preparation and sequencing. AK developed the tools used for the bioinformatic analysis and maintain the ARIES webserver. SM conceived the study, contributed to the scientific discussion and thoroughly revised the manuscript. All authors contributed to the article and approved the submitted version.

## Funding

The study was supported by intramural funds of the Istituto Superiore di Sanità.

## Conflict of interest

The authors declare that the research was conducted in the absence of any commercial or financial relationships that could be construed as a potential conflict of interest.

## Publisher’s note

All claims expressed in this article are solely those of the authors and do not necessarily represent those of their affiliated organizations, or those of the publisher, the editors and the reviewers. Any product that may be evaluated in this article, or claim that may be made by its manufacturer, is not guaranteed or endorsed by the publisher.

## Supplementary material

Supplementary Table 1 For each tested strain, the table includes the statistics of the *rpoS’* gene expression.

Supplementary Table 2 For each tested strain, the table includes the statistics of the assembled contigs, details on sequencing technology used and the virulence genes content of the genomes. Highlighted in yellow the *astA* gene’ allele encoding a thermostable toxin not present in the other ECORI strain.
